# Rheumatoid arthritis fibroblast-like synoviocytes show the upregulation of myeloid cell specific transcription factor PU.1 and B cell specific transcriptional co-activator OBF-1, and express functional BCMA

**DOI:** 10.1186/ar3629

**Published:** 2012-02-09

**Authors:** Kenji Itoh, Katsuya Nagatani

**Affiliations:** 1 Department of Rheumatology, National Defense Medical College, Tokorozawa, Saitama, Japan; 2 Department of Rheumatology & Clinical Immunology, Jichi Medical University, Shimotsuke, Tochigi, Japan

## Objective

Fibroblast-like synoviocytes (FLS) are among the principal effector cells in the pathogenesis of rheumatoid arthritis (RA). This study shows the variety of stimulating effects of a proliferation-inducing ligand (APRIL), and its specific effect on the FLS in the affected RA synovium (RA-FLS).

## Results

A significantly higher level of soluble APRIL was detected in RA serum compared with in normal serum. Among the three receptors of APRIL tested, RA-FLS expressed only the B cell maturation antigen (BCMA), whereas the FLS in the affected osteoarthritis synovium (OA-FLS) expressed none of the receptors. Moreover, RA-FLS expressed transcription factor PU.1 and B cell specific-transcriptional co-activator OBF.1, which were normally expressed during myeloid and B-lymphoid cell development. The expression levels of PU.1 and OBF-1 were correlated with those of BCMA in RA-FLS. APRIL stimulated RA-FLS but not OA-FLS to produce interleukin (IL)-6, tumor necrosis factor (TNF)-α, IL-1β and APRIL itself. APRIL also enhanced the receptor activator of nuclear factor kappa B ligand (RANKL) expression in RA-FLS. Moreover, APRIL enhanced the cell-cycle progression of RA-FLS. Neutralization of APRIL by BCMA-Fc fusion protein attenuated all these stimulating effects of APRIL on RA-FLS.

## Conclusions

RA-FLS express BCMA, and are stimulated by APRIL. These results provide evidence that APRIL is one of the main regulators in the pathogenesis of RA. Epigenetic regulation of BCMA transcription in RA-FLS might contribute to the underlying mechanisms of this condition.

